# Only an integrated approach across academia, enterprise, governments, and global agencies can tackle the public health impact of climate change

**DOI:** 10.3402/gha.v6i0.20513

**Published:** 2013-04-03

**Authors:** Gunhild A. Stordalen, Joacim Rocklöv, Maria Nilsson, Peter Byass

**Affiliations:** 1 Stordalen Foundation, Oslo, Norway; 2 Umeå Centre for Global Health Research, Umeå University, Umeå, Sweden

**Keywords:** climate change, public health, academia, research, enterprise, government, international agencies, human health

## Abstract

**Background:**

Despite considerable global attention to the issues of climate change, relatively little priority has been given to the likely effects on human health of current and future changes in the global climate. We identify three major societal determinants that influence the impact of climate change on human health, namely the application of scholarship and knowledge; economic and commercial considerations; and actions of governments and global agencies.

**Discussion:**

The three major areas are each discussed in terms of the ways in which they facilitate and frustrate attempts to protect human health from the effects of climate change. Academia still pays very little attention to the effects of climate on health in poorer countries. Enterprise is starting to recognise that healthy commerce depends on healthy people, and so climate change presents long-term threats if it compromises health. Governments and international agencies are very active, but often face immovable vested interests in other sectors. Overall, there tends to be too little interaction between the three areas, and this means that potential synergies and co-benefits are not always realised.

**Conclusion:**

More attention from academia, enterprise, and international agencies needs to be given to the potential threats the climate change presents to human health. However, there needs to also be much closer collaboration between all three areas in order to capitalise on possible synergies that can be achieved between them.

The public health impact of climate change has been under-researched and not well communicated within the overall quest to understand the consequences of current and future variations in the global climate. Thus there is an urgent need to better understand the health challenges posed by changes in weather and climate in order to protect the health of current and future generations. It must also be recognised that global changes in climate require local health protection actions – solutions do not come on a one-size-fits-all basis.

Climate, weather, and human health are interrelated in complex ways within the earth's biosphere, and these interactions are strongly influenced by various aspects of human society (1). The scope of these societal determinants ranges from the application of scholarship and knowledge, through economic and commercial considerations, to the actions of governments and global agencies. In this discussion article, we try to understand some of the ways in which this range of societal determinants both fails and succeeds in protecting human health from the short-term effects of weather (floods, droughts, storms, heat, etc.) and the long-term aggregation of changes in weather patterns, namely climate change. In addition to these macro-level societal determinants, climate and weather also act as individual-level social determinants of health, in that disadvantaged or poorer people are likely to be less able to manage and mitigate possible effects of weather on their health.

We argue that to achieve useful action across the range of societal determinants, public health must be centre stage. The win-win effects of climate and health co-benefits (whereby, for example, increased physical activity can lead to reductions in disease risk and carbon footprint) need to be nurtured and harvested across all sectors.

## The role of academia in climate and health

Scientific and academic insights into the complexities of climate, weather, and health constitute a fundamental building block of the societal response to the human consequences of climate change. However, the large interdisciplinary range encompassed by these issues is itself challenging. Conventional academic institutions tend to be rather vertically divided, and so bringing together specialists in fields such as meteorology, climatology, environment, demography, health, epidemiology, economics, sociology, management, and politics – such that all have joint ownership of the science – is rare. What has happened more commonly is that established specialist groups in, for example, climate, have grown in-house subsidiary groups in, for example, health, of which the ‘ownership’ is largely retained by the original group. However, there are good examples of collaborations between academia, governments, and other agencies. In Europe, warning systems for heat waves and climate-driven infectious disease threats have been developed (2, 3), but for much of the world this is not yet the case.

Climate science typically uses and generates incredibly large volumes of data that may be relatively impenetrable to other disciplines. Health science also handles large volumes of data, but the global coverage and quality of these data tend to vary widely, often in proportion to economic levels (4). This is a particular problem for science at the climate and health interface, since some of the populations most at risk from the potential consequences of climate change are found in regions of economic and health data deprivation, such as Africa. Thus, in the relatively few African locations where detailed longitudinal health data are available, it is important to analyse these against corresponding weather data (5–8).

**Fig. 1:**
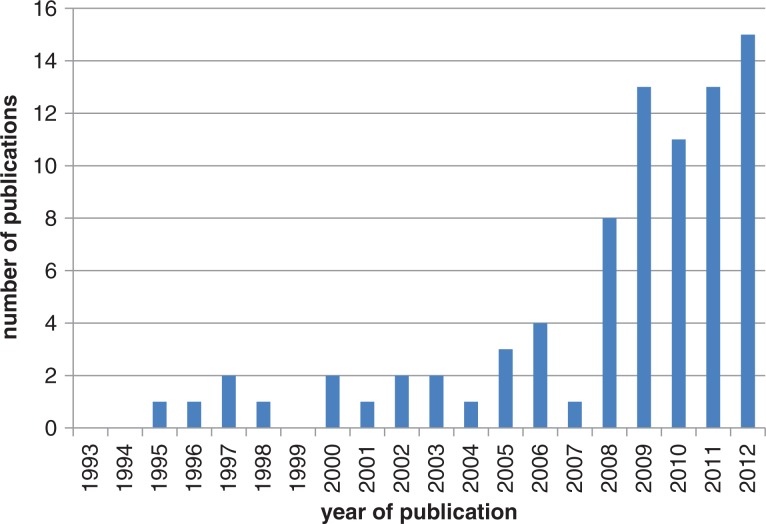
Number of citations in PubMed by year for the search terms (‘climate change’ and ‘health’ and ‘Africa’) over a 20-year period.

The global distribution of science linking climate and health remains problematic. We showed previously that up to mid-2009 only 0.002% of PubMed citations with keywords ‘health’, ‘Africa’ and ‘climate change’ included all three terms (9). From then up to the end of 2012, this proportion increased to 0.017%, even though the absolute numbers remain low, as shown in Fig. 1. Although this is a very crude metric, it shows both the paucity of research in this key area and increasing interest in it.

Table 1 shows global variations in academic output on health and climate. Not surprisingly, Oceania is well represented given the vulnerable position of various small island states, particularly when considered on a per-population basis. However, the larger vulnerable populations of Africa and Asia remain under-represented in research. Moreover, less than half of the papers from Africa included authorship from African institutions, reflecting an acute lack of human capacity in that region.

**Table 1:** Publications indexed in PubMed under the topics ‘climate change’ and ‘health’ by world regions and publication type

	Europe	Africa	Asia	Americas	Oceania
All publications	441	141	235	575	151
% of all publications	29%	9%	15%	37%	10%
Reviews	73	33	44	84	48
Reviews as % of all publications	17%	23%	19%	15%	32%
Population (millions)^a^	739	1,072	4,157	942	37
% of all population	11%	15%	60%	14%	<0.1%
Publications per 10 million population	5.9	1.3	0.6	6.1	40.8

a Source: Population Reference Bureau: 2010 World Population Data Sheet.

Perhaps partly because of the shortcomings of conventional academia in engaging with climate and health on a global basis, governmental and international bodies have to some extent taken on responsibilities for generating knowledge in this important area. The best known example is the Intergovernmental Panel on Climate Change (IPCC, www.ipcc.ch), a scientific body under the auspices of the United Nations, which has co-opted and co-ordinated large numbers of researchers to generate evidence on climate change. Its stated aim is to be ‘policy-relevant and yet policy-neutral, never policy-prescriptive’. The IPCC reports started naturally from climatological assessment and later incorporated societal aspects of climate change, including effects on health. This meant that climatologists were instrumental in outlining climate and health research in collaboration with health scientists, and the research and publications coming out of these efforts had obvious climatological perspectives. Such perspectives were not necessarily optimal – for example, in terms of the timescale considered – for the public health sector and much work still lay ahead to better integrate climate change and health research. Many climatological studies failed to make future projections of societal determinants, even though these are modifiers for health effects. The direct effects of climate change on human health have therefore been underplayed in the academic arena, and consequently often been overlooked in policy making.

## The role of enterprise in climate and health

The commercial implications behind climate and health issues have been relatively slow in developing, not least because most of the knowledge and evidence on climate and health has been generated in the public sector, where many academics and policy makers do not instinctively see things from a private sector perspective.

The concept of climate and health co-benefits (such as cycling to improve fitness and reduce carbon emissions) originated from public sector thinking, but carries important implications for the private sector. Health benefits of particular strategies, as advised by trusted health professionals, can be a way into health and climate co-benefits (10). For the private sector, there are inevitable consequences of any kind of widespread behaviour change. Thus if, for example, health professionals advocate cycling as a health and climate co-benefit, the bicycle industry could benefit commercially. At the same time, the car industry may be disadvantaged.

Increased active transportation (walking (including to and from public transport stops) and cycling) has been demonstrated to confer health benefits, including reductions in cardiovascular disease, mental disorders, and some cancers (11, 12). As well as the economic implications for the transport industry, in some settings, where medicine and health insurance are part of the private sector, there may also be important commercial implications for the health sector. Higher rates of public transportation also lead to lower rates of accidents and injuries, with consequent reductions in associated financial and health losses (13, 14).

A complex set of relationships exists between food, health, climate, and agriculture. The growing global population and increasing environmental and sustainability concerns call for safe, nutritious, affordable, and healthy food. Intensified competition for land use and shifting dietary patterns globally, including significant increases in meat consumption, are rapidly creating new supply/demand scenarios. Consequently the food industry is increasingly emphasising reducing wastage, energy, and water consumption at all stages of the supply chain. Links between food quality, human health, and the environment must be acted upon in optimizing food systems and maintaining the competitiveness of the food industry in a changing world.

Furthermore, to remain competitive, the food industry must respond to changing consumer behaviour and preferences. The impact of food choices on the health and well-being of citizens has far-reaching implications. There are complex socio-economic and cultural issues surrounding consumers’ perception of and attitudes towards food, including traditional foods, societal and cultural trends, and other factors related to food choice and consumer access to food. Research is needed to improve our understanding about food, food choices, and the impacts of those choices for example on health, on society, on the environment, and on competitiveness and sustainability.

Not all foodstuffs, and hence diets, carry similar implications for greenhouse gas (GHG) emissions. Meat and fish production in particular are associated with much higher emissions than other food sectors (15), and Faber et al. (16) have argued that shifts towards more vegetarian diets would achieve major carbon reductions. From a health perspective, the links between high consumption of red and processed meat and increased risks of death from cardiovascular disease and cancer have been shown (17), making the co-benefits of reduced consumption for both climate and health substantial. But again, were this to happen on a large scale, there would be major commercial consequences within the food industry.

The traditional view of the private sector has been that only the bottom line (i.e. maximised profitability) matters. This is a concept that is changing rapidly due to various influences, and the concept of multiple bottom lines has emerged (18). In addition to overall profitability, these may include results on environmental sustainability and workforce well-being. Perhaps an additional bottom line, of results in terms of population health benefits of climate-friendly policies, may emerge as inherent relationships between climate and health become clearer.

In a challenging global economy, a growing number of corporations are turning to sustainability to keep competitive according to a recent MIT report (19). The study found that two-thirds of companies see sustainability as a competitive necessity in today's marketplace, a substantial increase from the previous year. The data, which include responses from 2,874 executives in 113 countries, suggest that the sustainability movement is nearing a tipping point, the point at which a substantial portion of companies are not only seeing the need for sustainable business practices, but are also deriving financial benefits from these acts, the report concludes. The Global 100 Most Sustainable Corporations outperformed the general market by 11% from 2005.

Furthermore, a recent UNEP report (20) showed that business cannot afford to ignore the benefits that switching to a green economy will bring. The ability of the private sector to embrace ideas, innovate, conceptualise, and develop solutions in the form of new products and services is something needed at all levels of society to achieve a green economy transformation, and thereby indirectly achieve population health benefits.

Green economy benefits to business include more resilient supply chains, new investment opportunities, increased consumer demand for sustainable goods and services, sales growth and job creation, reduced dependency on natural resources, and mitigation against the negative financial risks of environmental impact. Consumer interest in sustainable products is progressing from early fears about assumed higher costs to a trend in which positive environmental and social elements are becoming an inherent part of product quality. Recent survey results (21, 22) suggest that in future business-to-business (B2B) and business-to-consumer (B2C) transactions will carry expectations of products being environmentally and socially responsible.

It is clear that the majority of individuals may be reluctant to change behaviour for the sake of the planet, but may be more inclined to make relatively simple changes which are associated with tangible benefits such as better health. Similarly, at the corporate level businesses are more likely to change in ways that are motivated by improvements in their profitability, image, and consumer appeal, rather than purely on grounds of environmental sustainability. Thus the co-benefits concept is also crucial for the private sector – making it easy for businesses and their customers to change in ways that bring wider and longer-term advantages, by focusing on direct and tangible benefits.

## The role of governments and global agencies in climate and health

The response of national governments to the important issue of climate and health has varied. In some cases, for example, Australia, there has been very explicit recognition of the physical and mental health implications of global climate change (23). Many other countries have largely ignored the issue. Regional agencies such as World Health Organisation (WHO) Europe have also been active in assessing health impacts from policies to mitigate climate change, suggesting that these can be found in the transport, housing, food and agriculture, and energy sectors (24).

Most countries engage with the global UN Framework Convention on Climate Change (UNFCCC), which addresses health in the first and fourth article of the convention by requiring parties to ‘Take climate change considerations into account… with a view to minimizing adverse effects… on public health’ (25). So protecting health is one of the United Nations’ motives for engaging in work to counteract potential climate change. In the IPCC Fourth Assessment Report, there was increased emphasis on climate change impacts on human health (26). Despite this, human health has received little attention so far in the UNFCCC process. But there are some signs that a change is underway; one example is increasingly common references to health in reports from national parties (countries). An increasing number of international and national NGOs are drawing attention to health impacts in side-events to the Conference of Parties (COP) meetings. At COP 17 in Durban in 2011, more than 10 side-events addressed health with one of them being the first Global Health Summit organised by international NGOs and WHO, bringing key actors from the health sector together (27). At COP 18 in Doha in 2012, health and medical organisations across the world agreed on a declaration calling for the protection and promotion of human health as a priority in global and national policy responses to climate change (28). The declaration was widely disseminated during the COP meeting. A limited study of accredited participants at COP 16 acknowledged that human health should be an important factor in the UNFCCC negotiations (29).

The WHO, the directing and coordinating authority for health within the United Nations system, adopted a resolution in 2008 (61.19) on climate change and health giving a framework for action for governments (30). The resolution stresses the health sector's responsibility to increase efforts on climate change adaptation projects, to raise awareness of climate change health impacts at national and international levels and to strengthen political attention and awareness. WHO consulted with member states and prepared a work plan, which was adopted in 2009. At COP 18, WHO emphasised the view that health co-benefits should be guiding decisions on policies for adaptation and mitigation from climate change. However, whether this view will be taken on board by the wider climate community, and invested in by the enterprise sector, remains to be seen.

Is it realistic to expect that global agencies like UNFCCC have the power to change the way the world operates and to transform the risks to human health of climate change? After each COP meeting, the global media regularly report on the UN having failed again. This kind of reporting can have a negative impact by enhancing despair and distrust of the process, with possible far-reaching consequences. Climate change is extremely complex in itself, with feedback between the climate system, ecosystem, and the human system – as well as the negotiation process in which 194 states with very different environmental, political, and economic conditions and agendas try to find agreement. In addition, there is a huge spectrum of countries from the most to the least powerful, and the most to the least vulnerable. There is a clear need to find greater consensus between the knowledge base, commercial considerations, national interests, and the UN agencies if damage to health from climate change is not going to out-run potential protective strategies.

In parallel with the continued negotiations on binding GHG agreements, which is obviously difficult, a strategically important task for the UN is to focus on the implementation of mechanisms and programmes to enhance more immediate actions. The world needs to see positive examples; the champions, pilots, and role models. Those who engage and act need to get the visibility they deserve as good examples.

Christiana Figueres, the Executive Secretary of the UNFCCC, during the COP 18 meeting, said that ‘we need to change the mood of the world’ when she addressed the civil society groups. Key components of any change are motivation and confidence in that change is possible. In the climate efforts, those components are needed, involving as many players as possible: a top-down and bottom-up approach with stakeholders at all levels.

Just before the Doha meeting, the World Bank reported that the world is most likely heading for an increase of 3.5–4.0°C from pre-industrial climate levels, given current pledges and commitments from countries in the UNFCCC process (31). This would bring considerable health risks to some of the world's most vulnerable people. The report received far-reaching attention in international media and was widely referenced. While this provided additional incentives to reach a GHG agreement, it also highlighted the considerable vested interests against so doing. This highlights some of the considerable difficulties facing governmental and international agencies in bringing about change.

## Conclusions

The academic, commercial, and political influences on climate change and health are considerable. In some aspects, they act synergistically, for example, when knowledge is used for successful policy making. In other situations, there can be substantial power battles, for example, when evidence calls policy into question or challenges vested economic interests.

None of the three major societal dimensions we have discussed can claim to have a neutral role in terms of possible future threats to human health from climate change. All three areas have the potential to do better: there is a need for more evidence, with a wider global spread; commercial operations need to be increasingly climate-friendly and can do so without damaging profitability; and governments and agencies need to take stronger stands on important mitigation steps. However, improvements in each area separately will not be sufficient: the world needs to capitalise on synergies arising out of integrating all three domains to achieve maximum impact.

How to promote increasing integration across all these areas in practical ways that can lead to better public health outcomes in the face of the challenges of climate change remains a crucial question. Academic findings frequently need more interpretation into the realms of costs and profits for the enterprise sector, and statutory agencies, in interpreting research into policy, must explicitly include commercial implications. Achieving better integration is a matter of urgency, against the background of inexorable climate change.
